# Clinical aspects of palliative care in advanced Parkinson’s disease

**DOI:** 10.1186/1472-684X-11-20

**Published:** 2012-10-25

**Authors:** Johan Lokk, Ahmad Delbari

**Affiliations:** 1 Department of Neurobiology, Care Sciences and Society, Division of Clinical Geriatric, Huddinge Hospital, R94, Karolinska Institute, Stockholm, Sweden, SE–141 86; 2 Iranian Research Center on Healthy Aging, Sabzevar University of Medical Sciences, Sabzevar, Iran

**Keywords:** Clinical aspects, End-stage, Palliative care, Parkinson’s disease

## Abstract

Parkinson's disease (PD) is one of the most common neurodegenerative disorders of the elderly population. Few therapeutic options are available for patients with PD requiring palliative care. Treatment of the early stages of PD is entirely different from later stages. During the later stages, the palliative care model is introduced to provide the patient with comfort and support. Early palliative care in PD requires minimization of dyskinesias and decreasing occurrence of motor and non-motor off times in an effort to maximize independent motor function. In the later stages, the focus of treatment shifts to treating the predominant non-motor symptoms and having a more supportive and palliative nature. The purpose of this review is to provide a summary of the palliative care management issues and palliative care management options of end-stage PD patients.

## Review

Parkinson's disease (PD) is one of the most common neurodegenerative disorders of the elderly population with an average age of onset of 60 years of age [1]. PD affects 17.4 per 100,000 of individuals between the ages of 50-59 and 93.1 people per 100,000 of individuals between the ages of 70-79 [2,3]. The average patient lives 15 years from the time of diagnosis until death [4]. PD is currently assessed as more of a systemic brain disease with many different dysfunctional or dying neuronal cell systems thus affecting different transmittor substances and not only a loss of specific dopaminergic neurons in the substantia nigra. As PD progresses, there is a also presence of Lewy bodies within the cytoplasm of the affected dopaminergic neurons and a hierarchical spread of these within the brain from the medulla oblongata through the substantia nigra to the cortex. The PD course could be described as passing through a trajectory of phases starting with a “honey-moon” phase with an almost complete symptom relief due to pharmacological treatment, followed by a motor complication phase, neuropsychiatric phase, and ending up in the palliative phase. However, this is only a way to describe the inevitable progression of the disease as many symptoms may occur during all stages of the disorder. Unfortunately, few therapeutic options are available for PD patients who have progressed to more advanced disease [5]. There are several therapeutic options for advanced PD patients including intraduodenal levodopa infusion, apomorphine infusion and deep brain stimulation. They have different profiles and are addressing different PD phenotypes. However, end-stage PD patients, especially if they are cognitively impaired as they often are, exclude them from these therapies. Medical interventions are largely ineffective in preventing the inevitable progression of PD since there is always also a possibility of an intercurrent illness. Additionally, there is a high probability of PD patients to become disabled and dependent [6]. PD patients who do not respond to standard treatments require multidisciplinary care that include elements of traditional medicine and holistic care. In this multidisciplinary care model, treatment of common non-motor symptoms like pain and depression are complemented with holistic therapies including psychological, social and spiritual problems that might arise [7].

According to the World Health Organization (2005) palliative care is to provide comfort and support for people who are facing life-threatening illnesses in order to improve the quality of their lives as well as those of their families [8]. This can be done by identifying the illness, assessing, and treating the pain as well as other physical, psychological and spiritual problems that might cause suffering.

In order to maximize the quality of life for these patients, the palliative care must include a team of medical providers, nurses, as well as additional caregivers [9]. Such an approach serves to provide traditional medical therapies, emotional and spiritual support while preserving patient autonomy and dignity as they manage their disease. Treatment of the early stages of PD is entirely different from later stages. Early treatment is geared towards prevention, symptom relief enhancement of survival, and prevention of motor symptoms. During the later stages, the palliative care model is introduced to provide the patient with comfort and support. In the later stages, however, the focus of treatment shifts to treating non-motor symptoms and having a more supportive and palliative nature. That is why, as the patient approaches the end-stage of the illness, the main goal of both the patient and the physician becomes management of motor and non-motor impediments according to the principles of palliative care. It is important to take into account that symptom control care includes the preserving of autonomy as well as stress relief. A holistic approach must be applied from the moment of diagnosis until the end of a patient’s life and should not only be reserved for the stage of bereavement.

The purpose of this review is to provide a short summary of some of the palliative care issues of end-stage PD from a clinical and pragmatic point of view and discusses how patients can be treated through the application of common principles of palliative care management. References for this review were recognized through search in PubMed for potentially relevant articles with the search terms of “Parkinson’s disease”, “palliative care”, and “advanced Parkinson’s disease. The review is focusing on the medical management of motor and non-motor manifestations and concentrates on end-stage PD patients.

### Pharmacologic issues at end-stage Parkinson's disease

As PD progresses there are fewer dopaminergic neurons in the substantia nigra as well as a progressively lower capacity to store exogenous levodopa and convert it to dopamine for storage and release in the remaining neurons. Additionally, as the dose requirements of levodopa increase, the patient's functioning is inhibited before his next dose of medication [10]. This usually takes place 2–4 hours after a levodopa dose and may appear as sensory (pain, paraesthesiae), psychiatric (paranoia, anxiety, hallucinations, depression) or autonomic (sweating, belching, constipation, tachycardia, or breathlessness) symptom, or progression of motor symptoms or dystonia [11]. This is called “end of dose wearing off”. Wearing off has not been fully explored, but may be connected to pharmacokinetic changes, and shortening of striatal levodopa half-life resulting from progressive degeneration of nigrostriatal dopaminergic terminals [12]. To treat motor symptoms it is necessary to deliver medication effectively. Patients with advanced PD often notice that protein-rich meals will diminish the effectiveness of levodopa doses [13]. This can be alleviated by reducing protein intake during daytime and limiting protein intake at night to 40 grams [9]. There are some strategies to Improve absorption of Levodopa include: advise patients to take levodopa before meals, on an empty stomach [14], advise patients to avoid protein-rich meals and bulk-forming foods [15], advise patients against lying down, and encourage them to move around, after ingesting a dose [16], and advise patients to mix levodopa, crushed into a powder, into sparkling liquid [17].

As the disease progresses, current levodopa doses give hyperkinesia due to the narrowing of the therapeutic window. In addition, patients on polypharmacy regimens are often on other anti PD drugs that commonly elicit side effects like hallucinations and/or dyskinesia. This implies that the patient often must have monotherapy with levodopa as this drug is least prone to have side effects and that the doses must be lower and carefully monitored.

### Motor complications

Motor complications in PD consists of motor fluctuations and dyskinesia most probably as a result of the pharmacological treatment. These can be either excessive hypokinesia (e.g. freezing, rigidity, increasing off times, dysphagia, dysarthria, and respiratory compromise) or excessive hyperkinesia (e.g. choreiform and dystonic dyskinesia). Motor complications decrease the patient's quality of life in many ways as they affect emotional health, decrease mobility, decrease independence for activities of daily living, and cause social stigma [18]. Maintenance of independent motor function is the primary goal of treatment of early and later stages of PD. Such a strategy allows the patient to remain independently mobile for as long as is possible, and greatly improves quality of life. The focus of treating these motor complications in end-stage PD needs to make the patient as independent as possible for as long as possible by increasing the time with no dyskinesias and decreasing occurrence of motor and non-motor off times.

### Hypokinesia/akinesia

Among individuals with PD, gait disorders are one of the most common factors that affect independence and quality of life [19]. Debilitating hypokinesia as a type of motor fluctuation is one of the most common signs of end-stage PD. In these individuals, episodes of hypokinesia can occur many times per day and these events are typically associated with either a failure to respond or the “off” phase of dopaminergic treatment [9]. Frequent dosing of short-acting levodopa/carbidopa every 3–4 hours coupled with the catechol-o-methyl transferase (COMT) inhibitors, are currently the best therapy to minimize episodes of hypokinesia. This regimen causes the least variation of levodopa in blood levels, with less off-time, more on-time, and better quality of life. The COMT-inhibitor tolcapone has both central and peripheral effects on the dopaminergic metabolism in contrast to the COMT-inhibitor entacapone, which only acts peripherally. Tolcapone is particularly indicated in advanced patients where the otherwise most commonly used entacapone is no longer effective [20]. Dopamine agonists are another therapeutic option although they carry the risk of worsened cognition at the end of life [21]. Additionally, longer-acting sustained-release carbidopa/levodopa regimens can be used during sleeping hours if awakenings are accompanied by immobility [22].

### Dyskinesia

Among the major complications of managing PD is the presence of dyskinesia. Dyskinesias consist of abnormal movements (e.g. movement of the head, neck and limbs) that are both debilitating, physically tiring, and embarrassing. Several reports show that the rate of this problem vary greatly, ranging from 19 to 80% in PD patients [23]. Dyskinesias in end-stage PD are more frequent [24] and are likely to be a consequence of long-term levodopa therapy [25]. A recent study showed that PD patients treated with levodopa for 4–6 years have a 40% likelihood of experiencing dyskinesia [26]. Painful and debilitating dyskinesia are less common today than 10 years ago due to more cautious, careful and individualized anti-PD therapy. Lower doses of levodopa, earlier introduction of other anti-PD agents have contributed to this, However, once dyskinesias occur, lowering of dopaminergic therapy, adding inhibitors of MAO-B or COMT as well as adding amantadine could have some effect. However, dyskinesias don't seem to be a major problem when the patients get demented.

### Dystonia

Dystonia as a neurological movement disorder occurs in untreated PD patients.

The treatment of dystonia varies based upon clinical presentation. Early morning dystonia, a symptom of overnight wearing off, may respond to nocturnal long-acting dopaminergic agents. In contrast, peak-dose dystonia, that occurs during the day, may respond to reduced dose of dopaminergic medications, given more frequently in smaller doses [9]. EMG guided injections of botulinum toxins can be used to treat focal dystonia of a single muscle [27]. Anticholinergics, baclofen and benzodiazepines are regularly used with caution due to possible cognitive side effects in the end-stage PD patient [9]. The use of botulinum toxin (BT) is increasing in PD patients when treating dystonia, spasms, urinary bladder dysfunction and drooling. Targeted injections of BT, often guided by EMG, can be tried in these conditions. BT only starts having an effect after three to four days. This effect will gradually increase till about three weeks after treatment. There is no permanent effect and the treatment needs to be repeated after 3-4 months.

### Freezing

PD patients can experience freezing of mobility through any movement, but it is most prominent and difficult when this freezing involves gait. Freezing is especially frequent when initiating gait (start hesitation) and when passing through tight spaces such as doorways [28]. Freezing can be the result of either too much or too little dopaminergic effect. “Off freezing” may react to changes in the aforementioned medications, while “on freezing” is often associated with end- stage disease and is typically difficult to handle [28]. Non-pharmacological treatments and tricks can be used in freezing conditions – auditory cueing by counting figures loudly or clapping hands can be tried as well as visual cues like drawing lines on the floor, using a cane or the light of a laser pointer. These procedures might eliminate or diminish freezing episodes However, these techniques may be associated with an increased risk of falling, which PD patients already are at risk at, and fall prevention is essential to avoid serious fractures or injuries to the head [9].

### Palliative care management of non-motor complications

Patients with PD can develop non-motor manifestations, categorized into autonomic dysfunction, cognitive impairment, neuropsychiatric disorders, and sleep disturbances. At the end-stage of PD, non-motor symptoms become more common and can become the most prominent medical problem, leading to increasing decline in quality of life both for patient as well as increasing caregiver burden [29]. Non-motor symptoms occur in up to 50% of PD patients especially in association with the medication “off” state and may become worse by anti-PD medications [30]. Almost one third of patients reports their non-motor symptoms to be at least as debilitating as their motor symptoms [30].

All patients with motor fluctuations face at least one non-motor problem during the off phase [30]. In end-stage of PD, dementia, psychosis, and falls become more complex to manage than the motor complications; as a result, managing non-motor aspects is important to increase quality of life and decrease the burden of illness [31]. Chaudhuri and co-workers, using a new 30-item non-motor symptom screening questionnaire (the “NMSQuest”), found noticeably high scores among PD patients for impaired taste/smell, impaired swallowing, weight loss, constipation, urinary urgency, forgetfulness, dribbling, sadness, hallucinations, anxiety, sexual dysfunction, falling, reduced concentration, daytime sleepiness, vivid dreams, and sweating [32].

### Psychiatric complications

#### Psychosis

In most cases, psychosis develops late in PD, often due to underlying dementia and as a result of anti PD medication use. Around 40% of PD patients develop dementia in the late stages of the disease, and in these, psychosis is common. Patients suffering from PD dementia and psychosis are more likely to be placed in a nursing home and are also at an increased mortality risk [33]. Intercurrent medical conditions like constipation, dehydration, electrolyte abnormalities, pneumonia or urinary tract infection, may be hidden causes to psychosis and should be investigated and treated appropriately before starting antipsychotic treatment. Psychosis usually develops late in PD, often due to underlying dementia and as a result of anti-PD medication. However, it can occur throughout the whole trajectory of the disease.

The first step for psychosis treatment is to discontinue or decrease likely offending agents in the hierarchic order of anticholinergics, MAO-B inhibitors (MAO-BI, amantadine, dopamine agonists, and eventually levodopa [34]. However, there is then a risk of the patient having more motor problems. The physician should resort to atypical antipsychotics as the only remedy in the event psychosis persists despite best efforts to eliminate or decrease anti-PD drugs as being important contributors. The atypical antipsychotics, clozapine and quetiapine are possibly the most effective treatments, while olanzapine probably does not improve psychosis [35]. Risperidone is not a true atypical antipsychotic and has been found to exacerbate PD motor symptoms [36]. In general, all these drugs should be prescribed in low doses with slow upwards titration in order not to cause side effects. Moreover, acetylcholinesterase inhibitors (AChEI) may have a beneficial effect on mild and moderate psychosis.

#### Depression

Depression and anxiety occur in up to 40% of all PD patients, possibly higher among end-stage patients with increasing motor complications [37]. Anxiety as well as depression also tend to be more frequent during off-periods and often getting better when the dopaminergic treatment is optimized thus having less pronounced and less frequent off-periods. They can both be present throughout the disorder. Depression in the PD population has the most severe negative impact on reported quality of life [38]. In late stage PD, it is essential to closely evaluate patients, with the assistance of family and other caregivers, to identify depression. Besides from non-pharmacological treatment, antidepressants are widely used especially the serotonin reuptake inhibitors (SSRIs) [39]. Other drugs are less advisable because they carry more risk of cognitive side effects, and this is especially true for tricyclic antidepressants. Anxiety is closely related to depression and it is found that 66% of PD patients with motor fluctuations experience anxiety, often associated with irritability.

#### Cognitive complications

Cognitive impairment is found to occur in up to 80% of patients after 8 years of disease duration [40]. There are many steps when treating dementia, the first step being discontinuing unnecessary psychotropic medications, including amantadine, anticholinergics, sedatives, MAO-BI, and tricyclic antidepressants. The anti-dementia drugs used in Alzheimer's disease (AD) can also be used in PD dementia (PDD) despite there are only sparse documentation of the effect in PDD. However, it is reasonable to believe, and clinical experience speaks for it, that all AChE-Is – donepezil, rivastigmine and galantamine –show similar effects in PDD as in AD [29]. In line with AD treatment, memantine can also be used either as monotherapy or in combination with AChE-I in order to have additive or synergistic effect. Randomized controlled trials showed that the AChEIs donepezil and rivastigmine moderately improved cognition in PD. After 24 weeks of follow-up, some cognitive and behavioral functional improvements were observed in patients who suffered from mild to moderate PD treated with rivastigmine, which now is approved for PD dementia treatment [41].

#### Sleep disturbance

Sleep disturbances affect up to 60% of PD patients [42]. Sleep disorders can result from motor-related aspects as well as from non-motor-related aspects and medications. Anticholinergic medications, MAO-BI, and dopaminergic drugs can worsen the condition and must be considered to be decreased or stopped. Low-dose nightly clonazepam or melatonin might be useful. Nocturnal dystonia and cramping may cause sleep fragmentation and could be treated by a dopamine agonist at bedtime [10]. Nocturia and pain must be treated accordingly. As a consequence of sleeping problems, daytime sleepiness may appear, sometimes worsened by the use of dopamine agonists.

#### Apathy

Apathy occurs in 16.5 [43] to 42% [44] of PD patients. It is common and a main feature in end-stage PD and causes problems for clinical management and care. It seems that apathy is associated with cognitive impairment and depression and these should first be properly treated [45]. Based upon current clinical evidence, it is not clear whether apathy is improved by levodopa treatment.

### Autonomic dysfunction

80% of PD patients suffer from autonomic dysfunction and it may be a cause of significant morbidity [46]. Autonomic symptom severity in patients with PD seems to correlate with older age, greater disease severity, psychiatric complications, sleep disorders, and higher doses of dopaminergic medication. Conspicuous dysautonomias in patients suffering from severe PD include bowel and bladder abnormalities, gastrointestinal disorders, orthostatic hypotension and sexual dysfunction.

#### Orthostatic hypotension

Moderate orthostatic hypotension occurs in at least 20% of patients, correlates with disease duration, and may be a result of PD medications or intrinsic to the disease [46]. Orthostathism may result in deteriorating cognitive function, gait instability, fatigue and generalized weakness [29]. Non-pharmacological treatment entails increased sodium intake, compression stockings, improved hydration, and raising the head of the bed on blocks to improve the endocrinological regulatory system [47]. Pharmacological treatments contain the use of the NSAIDs, the sympathomimetic etilefrine, the mineralocorticoid fludrocortisone or the adrenergic agonist midodrine [9]. Treatment with droxidopa, a precursor of norepinephrine, has been demonstrated to be effective, well tolerated and safe when used to treat orthostatic hypotension in PD. A dose of 200 mg - 2,000 mg of droxidopa has been shown to increase standing blood pressure and standing ability in PD patients with orthostatic hypotension [48].

#### Gastrointestinal disorders

Constipation occurs commonly in PD patients [49] and is caused by intrinsic disease factors, medications, and reduced fluid intake [50]. In late-stage disease, constipation can be severe, particularly in cases where the patient is immobilized. It is one of the most common problems and can cause extreme discomfort [51]. Constipation is usually treated with stool softeners, lactulose, laxatives, increased water intake, dietary bulk, or exercise [34]. In all cases, anticholinergic agents should be discontinued [29]. Laxatives are important in preventing and managing constipation, but bulking agents, faecal softeners, osmotic agents and stimulant laxatives may be associated with complications like abdominal pain or diarrhea, especially after prolonged use [6]. Laxatives may not always be necessary if other remedies, e.g. dietary modification, can be used, assuming the patient is eating enough. It is better to give laxatives once or twice-weekly, so avoid daily use of stimulant laxatives [6].

#### Urologic dysfunction

Bladder dysfunction occurs in up to 70% of patients and is characterized by detrusor muscle hyperactivity, which leads to nocturia, urgency and increased urinary frequency [34,46]. It is imperative to rule out other causes of urologic dysfunction (urinary tract infection, prostate hypertrophy for men and stress incontinence for women) [29]. Patients should be particularly questioned about urinary symptoms and, when having urinary problems, instructed how to use toileting schedules pads and taught about fluid restriction at evenings, with botulinum injections into the prostate or bladder wall. If the dysfunction is due to detrusor muscle hyperactivity, it may be helpful to treat it with the antimuscarinic agents tolterodine or oxybutynin, however with caution due to the risk of impairing the mental state [10]. Tricyclic antidepressants with anticholinergic properties can also be useful by reducing bladder irritability [29]. Botulinum injections into the prostate or bladder wall could be helpful in some patients [34]. A urinary sheath or catheter is also an option if the dysfunction is highly significant and troublesome.

#### Pain

Pain occurs in up to 50% of PD patients, occurs throughout the disease, and remains an underreported complication of end-stage PD [6]. It can stem from a number of factors including musculoskeletal causes, motor, and non-motor complications. The most common motor causes are limb rigidity and dystonia. The success of treatment depends on identifying the type of pain experienced. If the pain gets worse in the “off” state, the treatment then should aim to maintain the “on” state of medication. If the pain is unrelated to levodopa doses, it should be treated with general pain management protocols [29]. Camptocormia is a stooped posture with flexion of the thoracolumbar spine. It is exaggerated with standing and causes back pain and spasm. Patients may respond to botulinum toxin injections to the rectus abdominus, physical therapy, and “sensory tricks” [52]. For pain symptoms, opiates should be used with caution, taking into consideration the increased risk of constipation in the end-stage PD patient with coexisting gastroparesis. Often all that is required is careful adjustment of anti-PD medication. Simple analgesics for musculoskeletal pain and adjuvants such as gabapentin for neuropathic pain are options pending careful evaluation of pain history [10].

#### Dysphagia and respiratory failure

Dysphagia is a common problem of late-stage PD, occurring in up to 95% of individuals, and is associated with aspiration pneumonia. Dysphagia is the result of disruption in the process of coordinated contraction and relaxation of the masticatory muscles, tongue, pharynx, and esophagus. Dysphagia raises the probability of recurrent aspiration owing to impaired coordination of pharyngeal muscles. It may lead to aggravation of motor symptoms due to reduced capability of taking anti PD therapy orally [9]. Swallowing difficulty materializes most obviously when choking on liquids. This problem can often be alleviated by altered consistencies, safe swallowing techniques and hand feeding, taking enough time to do this properly to ease swallowing and reduce the risk of aspiration [53]. If PD patients are experiencing significant swallowing dysfunction despite the alleviating measures, they should consult a speech pathologist and undergo swallow exam study using fluoroscopy. Recently, Christopher Kenney et. al have shown that the rotigotine patch is effective, leading to improved swallowing function in dysfunctioning patients [54].

PEG feeding tubes can be considered if nutrition or hydration is in danger of becoming inadequate or weight loss exacerbates the general condition. However, PEG insertion should only occur after careful consideration of the risks and benefits with careful explanation of these to the family. In end-stage PD, it is unlikely that PEG placement will improve medical results, but it may delay the decline to reach non-medical goals. Because dysphagia is usually better in “on” time and worse in “off” time, treatment should aim to increase “on” periods by adjusting dopaminergic therapy [28]. In additon, training and rehabilitation interventions now focus on repetitive practice; this also applies to swallowing as much as to walking or dressing.

Respiratory failure normally stems from hypoventilation or vocal cord adduction, and could be helped by proper anti-PD medication together with speech therapy. In severe cases, Non Invasive Positive Pressure Ventilation (NIPPV) or tracheostomy with assisted ventilation is an option [9].

### Pressure ulcers

As the disease reaches advanced or terminal stages, the risk of soft tissue ulceration becomes significant [55]. The development of pressure ulcers can lead to a decrease of physical, emotional, social and mental quality of life [56]. Because of the altered body position in PD, the ulcers may occur in other places than bony prominences. Caregivers need training in risk factors and techniques for moving and handling the patients. Physical activity can help to prevent contractures and pain resulting from immobility [10]. Active or passive movements in bed-bound patients can help to prevent contractures, breathing exercises can help preventing hypostatic pneumonia and frequent position changes can help preventing pressure ulcers.

### Institutional care

While most PD patients will cope at home for many years often with help of family caregivers as well as professional caregivers, increasing disability, dependency, psychotic, or harmful behavioral symptoms can precipitate nursing home placement [57]. Psychiatric comorbidity may also often occur in caregivers themselves because of the struggle they have caring for the patient [58]. Caregiver burden in PD correlates with increasing patient dependency and disability, depression, hallucinations, and falls [59]. Institutional care might therefore function as a relief of responsibilities for caregivers.

The principal goals of institutional care are to prevent complications, minimize distress, maintain patient dignity and provide counseling and symptom relief [60]. In institutional care, life-prolonging therapy is gradually substituted by palliative measures as the disease progresses [5]. The patient and family are supported by the multidisciplinary skills of the hospice team through end-of-life care which includes bereavement services for families [61]. The issues of withholding and withdrawing life prolonging treatment, and best interests decision making as well as letting patients die should also be considered.

A multidisciplinary team approach can peacefully lead a patient from disability to death [9]. Earlier studies have shown that patients who received institutional care had improved pain management, fewer emergency care hospitalizations, fewer invasive procedures, and adequate end-of-life care [62]. Meeting all the needs of PD patients in a palliative state is a hallmark of institutional care with an overriding objective to let the patients end their life in a professional and comfortable environment.

## Conclusions

During the later stages, the palliative care model is introduced to provide the patient with comfort and support. The focus of treating motor complications in end-stage PD needs to increase the time with no dyskinesias and decreasing occurrence of motor and non-motor off times. In advanced PD patients, the focus of treatment shifts to treating the predominant non-motor symptoms and having a more supportive and palliative nature.

## Competing interests

The authors declare that they have no competing interests.

## Authors' contributions

Both authors contributed to this review. A.D was responsible for drafting the manuscript and J.L guided in drafting, was responsible for revising the review critically and approved the final version of the paper.

## Pre-publication history

The pre-publication history for this paper can be accessed here:

http://www.biomedcentral.com/1472-684X/11/20/prepub
